# Massive ovarian edema with fallopian tube torsion treated with transumbilical laparoendoscopic single-site surgery: a case report

**DOI:** 10.3389/fonc.2025.1475166

**Published:** 2025-10-01

**Authors:** Dan Mu, Yangmei Shen, Yali Chen

**Affiliations:** ^1^ Department of Obstetrics and Gynecology, West China Second University Hospital, Sichuan University, Chengdu, China; ^2^ Key Laboratory of Birth Defects and Related Diseases of Women and Children (Sichuan University), Ministry of Education, Sichuan University, Chengdu, China; ^3^ Department of Pathology, West China Second University Hospital, Sichuan University, Chengdu, China

**Keywords:** fallopian tube torsion, fertility-sparing surgery, massive ovarian edema, suspension surgery, transumbilical laparoendoscopic single-site surgery

## Abstract

Massive ovarian edema (MOE) is a rare benign condition that can occur at any age, and mainly affects women of childbearing age and prepubertal girls. Patients with MOE do not have specific signs and symptoms, and imaging may show cystic or solid masses. Therefore, it is often unclear preoperatively whether the mass is a benign or malignant ovarian tumor. This increases the possibility of salpingo-oophorectomy due to suspicion of malignancy which, in turn, affects the fertility of young women and alters their sex hormone levels. We report the case of a 14-year-old girl with three complete turns of torsion of both the right fallopian tube and right ovary, and enlargement of the right ovary without necrosis. We performed transumbilical laparoendoscopic single-site surgery (TU-LESS) for diagnosis and treatment. During surgery, an ovarian cyst was removed and sent for frozen section, revealing MOE. Fertility-sparing surgery was therefore performed and the affected ovary was suspended to prevent further torsion.

## Introduction

Massive ovarian edema (MOE) was first reported by Kalstone et al. in 1969 as a rare benign lesion, and defined by the World Health Organization as the accumulation of edematous fluid in the ovarian stroma separating the normal follicular structures (1, 2). The etiology of MOE is unclear, one hypothesis being that ovarian torsion affects venous and lymphatic drainage, leaving the arteries unaffected; this causes massive ovarian interstitial edema without necrosis. Cases of MOE without ovarian torsion have also been reported (3, 4). While MOE mainly occurs in women in their 20s and 30s (4), some studies have reported it in children and menopausal women (4–6). The most common symptoms in MOE patients include abdominal distension, an abdomen mass, infertility, and irregular vaginal bleeding. In cases of acute torsion of the ovary, patients experience abdominal pain. The preoperative diagnosis of MOE is challenging due to the absence of characteristic clinical manifestations and imaging features in patients. Ultrasound findings in most patients reveal a nonspecific solid mass, either tumor-like or with cystic components, which could not be differentiated from tumors (7). However, a previous study reported that solid tumor-like ovarian masses with multiple peripheral follicles, with or without endometrial hypertrophy on ultrasound, may indicate the presence of massive ovarian edema (8). Another study also reported that the most distinctive feature of MOE seen on magnetic resonance imaging (MRI) was the presence of multiple follicles surrounding an enlarged ovarian cortex (9). Although these imaging examinations serve as important indicators for the diagnosis of MOE, clear preoperative diagnosis remains challenging, ultimately requiring pathological results for its confirmation. In pathology, MOE often manifests as ovarian interstitial edema while preserving ovarian structure, with luteinized stromal cells observed in a few cases (7). Due to nonspecific imaging and its clinical features, MOE is rare and difficult to diagnose preoperatively; thus, it is easily misdiagnosed as a malignant tumor, leading to overtreatment.

**Figure 1:**
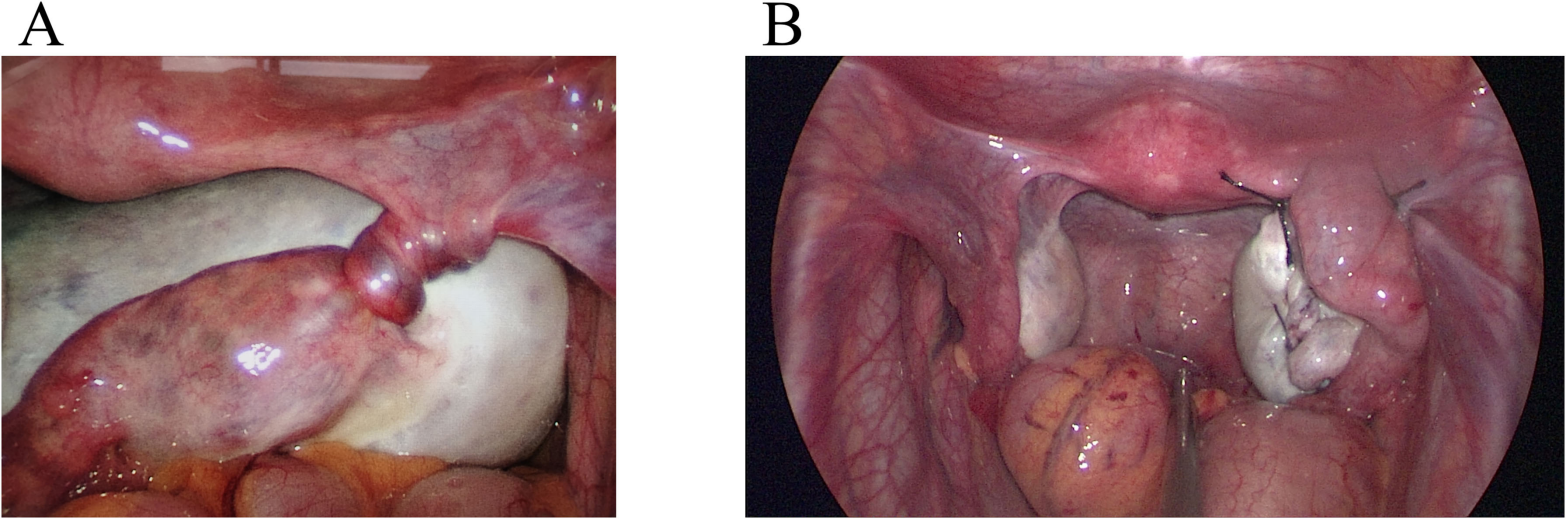
Laparoscopic findings. **(A)** The right fallopian tube was twisted 1080° before returning to normal anatomy. The right fallopian tube was thickened and the surface vessels were distended. **(B)** After reduction, the right ovarian cyst was removed, and the ovary was sutured and suspended. The ovaries were seen to be of normal size bilaterally.

Here, we report a case of MOE that was not diagnosed preoperatively. The diagnosis was suggested on intraoperative frozen section; therefore, we preserved fertility and performed ovarian suspension (**Figure 1**).

## Case description

**Figure 2:**
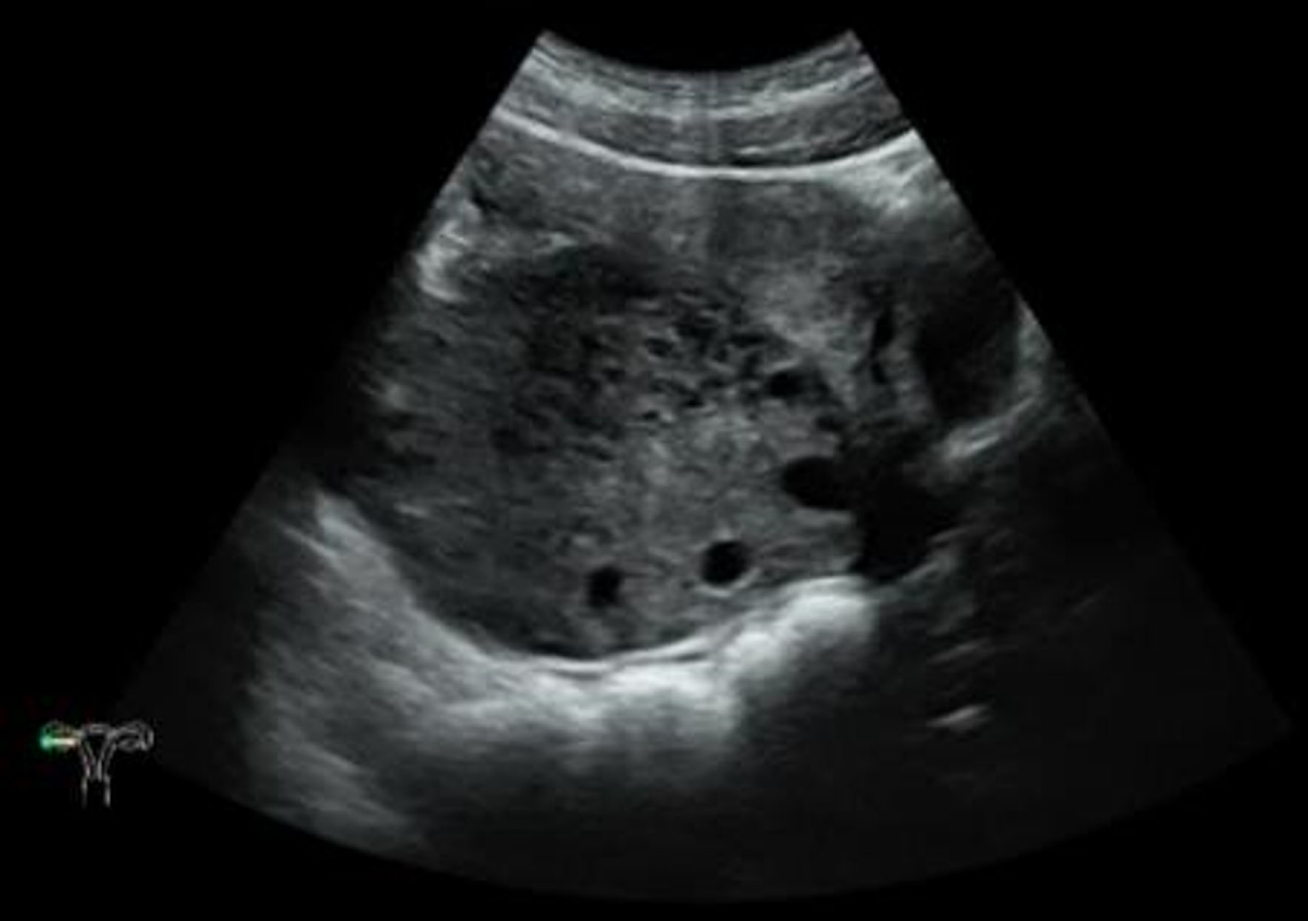
Transabdominal ultrasound findings. There was a heterogeneous, hypoechoic right adnexal mass with multiple anechoic areas.

**Figure 3:**
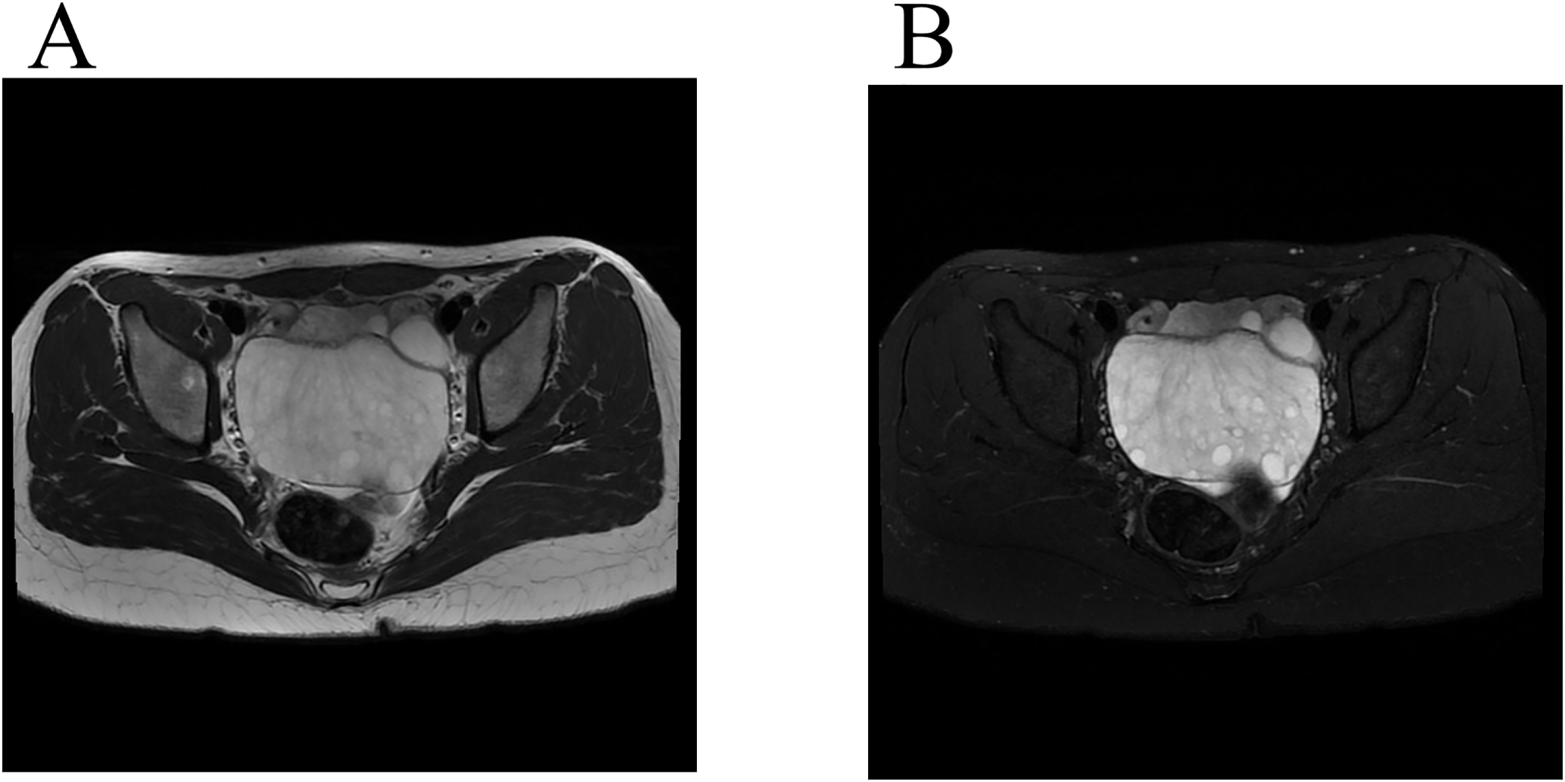
Magnetic resonance imaging findings. **(A)** A low-signal mass was seen on T2-weighted MR imaging (Axis). **(B)** A high-signal mass was seen on fat saturation T2-weighted imaging (Axis), and peripherally located follicles.

A female patient aged 14 years 7 months complained of mild lower abdominal pain and a prolonged menstrual cycle of more than 2 months. Menarche occurred at 14 years of age, with a menstrual period of 4 – 5 days and a cycle of 30 – 80 days. Physical examination revealed a widespread distribution of dense hair, including on the vulva, below the umbilicus, and around the anus. A large mass, approximately 10 cm in diameter, was palpated posterior to the uterus. Transabdominal ultrasound showed a heterogeneous, weakly echogenic right adnexal mass measuring approximately 8.6×5.9×9.0 cm, with multiple anechoic areas and blood flow signals (**Figure 2**). Magnetic resonance imaging (MRI) showed a mass measuring approximately 4.7×9.1×6.5 cm posterior to the uterus, which had a low signal on T1 weighted images and a heterogeneous high signal on T2 weighted images (**Figure 3**). Multiple small cystic shadows were observed. Tumor markers and sex hormone levels were within normal limits, including: ThCG<2.0 mIU/ml, CA125 7.3 U/ml, CA19-9 24.3 U/ml, CA15-3 7.0 U/ml, CEA <0.5 ng/ml, AFP 1.9 ng/ml. The patient underwent surgery via TU-LESS.

**Figure 4:**
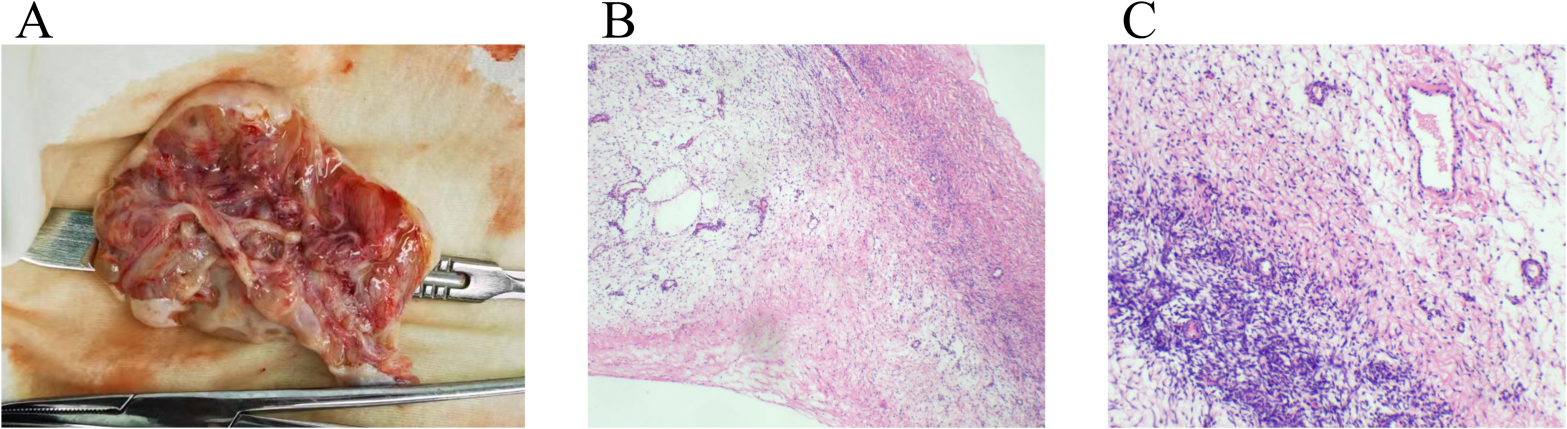
Pathological findings of excised ovarian cyst. **(A)** The ovary was cystic and solid, the thickness of the cyst wall was 0.2-0.4cm, the outer wall was smooth, and the inner wall was gray and edema. The focal area was multilocular, and the atrium contained clear fluid. **(B)** Ovarian subcortical fibrous stroma was edematous. The superficial cortex was not edematous(HE×40). **(C)** At high magnification, the ovarian cortex showed fibrointerstitial edema(HE×100).

Intraoperatively, the right fallopian tube and right ovary were torted with three complete turns, and the right fallopian tube was filled with blood vessels (**Figure 1**). The right ovary was enlarged, with a smooth, white, solid-cystic mass, approximately 10 cm in diameter; no blue-purple changes were observed. The uterus appeared normal in shape and size. The enlargement of the right ovary and torsion of the right adnexa were observed, with no obvious abnormalities detected in the left adnexa, liver, digestive tract, omentum, pelvic, and abdominal cavity. We placed the right ovary in a retrieval bag and removed the mass. The resected tissue showed multilocular edema, with the appearance of ovarian tissue with edema and deformation. There were dozens of small 0.3 – 3 cm cysts in the lesion, with clear gelatinous tissue and yellow gelatinous fluid. Two frozen sections of the excised ovarian lesions were obtained, showing MOE. The right ovary was intact and suspended with sutures and on the right round ligament. The postoperative pathology results confirmed MOE (**Figure 4**).

The patient was followed up postoperatively at the hospital’s outpatient clinic at one month and five months. Pelvic ultrasound revealed that both ovaries were normal in size, with no mass in the bilateral adnexa. Eleven months after surgery, a follow-up pelvic ultrasound revealed a slight enlargement of the right ovary, measuring 4.6*3.1*3.9cm, with multiple follicles (7-8 in one section), with the maximum measuring 1.5*1.2*1.7cm. These findings indicate a potential recurrence of right ovarian edema, requiring intensive monitoring.

## Discussion

MOE is a benign solid condition and can be either primary or secondary (4, 10). Secondary MOE is associated with Meig’s syndrome, retroperitoneal lymphoma, mature cystic teratoma, mucinous cystadenoma, serous cystadenoma, polycystic ovary syndrome, metastatic cancer, and use of ovulation induction drugs (11–16). There have been case reports of a small proportion of pregnant women developing MOE (17).

MOE tends to occur in the right ovary, Kanbour et al. (18) suggested this is due to higher pressure in the right ovarian vein, which drains directly into the inferior vena cava. However, the underlying etiology remains unclear. Most studies have considered impaired lymphatic and venous return from intermittent or partial torsion of the ovarian pedicle as a cause for MOE. In our patient, torsion of both the right fallopian tube and ovary, and frozen section suggesting edema of the ovarian stroma with dilated blood and lymphatic vessels, supports this theory. However, some patients do not have surgically confirmed torsion (3). This condition may result from the compression of the blood vessels or lymphatic vessels of the mesovarium by ovarian or para-ovarian lesions, obstructing drainage and leading to fluid accumulation (19). Diagnosis of MOE mostly occurs through surgical exploration, with ovarian lesions obtained during the operation and confirmation through pathological examination.

The clinical manifestations of MOE include acute abdominal or pelvic masses with or without lower abdominal pain, menstrual irregularities, infertility, virilization, hirsutism, and precocious puberty (19, 20). Our patient experienced mild lower abdominal pain, prolonged menstrual cycles, and hirsutism. Hirsutism is often associated with elevated androgen levels. Kalstone et al. proposed that luteinization might be triggered by mechanical stimulation from edematous fluid that stretches the stroma (1, 19). This suggests that patients with androgenic clinical signs may have excess secretion of androgens, which are produced by luteinized stromal cells in the edematous ovary (11). However, our patient had normal androgen levels, and no luteinization of stromal cells on histopathology; therefore, hirsutism may have been a normal physiological manifestation of puberty.

The symptoms of MOE are not unique. The present case showed that combining ultrasound with MRI images is helpful for preoperative diagnosis. On MRI, MOE appears hypointense or of medium intensity on T1 weighted images and homogeneous or heterogeneous hyperintensity on T2 weighted images (4). Other imaging findings of MOE include enlarged ovaries with interstitial edema, presence of multiple follicles in the peripheral ovarian cortex, and preservation of Doppler arterial flow (4, 8).

The imaging findings of our patient were consistent with those of other studies. These imaging findings can be used to diagnose MOE preoperatively. However, MOE is rare and difficult to differentiate from other ovarian diseases. Since Kalstone et al. first reported this disease in 1969, approximately 200 cases have been reported (21). The majority have been treated with salpingo-oophorectomy because of difficulty in diagnosing MOE pre- and intraoperatively and the potential for missing an ovarian malignancy (19, 22, 23).

In the patient, MRI did not reveal malignancy; however, transabdominal ultrasonography did not exclude the possibility of a germ cell tumor. Given the limited awareness of MOE, the condition was approached preoperatively as a potential malignant tumor. Considering the patient’s age (14 years), a laparoscopic exploration was prioritized to facilitate quicker physical recovery and minimize surgical trauma. During the procedure, the plan included assessing the mass shadow identified in the posterior uterus and converting to an open surgical approach if intraoperative findings or frozen section pathology suggested malignancy. This strategy allows for a minimally invasive initial intervention while maintaining the flexibility to address more complex surgical needs if necessary. Given concerns that removing the ovary and fallopian tube during TU-LESS would adversely impact the patient, frozen sections were performed, ultimately confirming the diagnosis of MOE. Ultimately, the ovary was preserved on the side of the lesion. MOE can be treated with conservative surgery; however, removal of diseased ovarian tissue, pathological evaluation, and exclusion of contralateral ovarian disease is necessary.

In conclusion, given the rarity of MOE and its limited understanding, the condition is easily misdiagnosed as a malignant ovarian tumor. While treatment often involves ovariosalpingectomy, this is considered overtreatment. Preoperatively, MOE should be suspected based on imaging findings and diagnosed using intraoperative pathology in order to avoid overtreatment and prioritize fertility preservation.

## Data Availability

The original contributions presented in the study are included in the article/supplementary material. Further inquiries can be directed to the corresponding author.
